# CKIP-1 Is an Intrinsic Negative Regulator of T-Cell Activation through an Interaction with CARMA1

**DOI:** 10.1371/journal.pone.0085762

**Published:** 2014-01-17

**Authors:** Takashi Sakamoto, Masayuki Kobayashi, Kohei Tada, Masanobu Shinohara, Katsuhiro Io, Kayoko Nagata, Fumie Iwai, Yoko Takiuchi, Yasuyuki Arai, Kouhei Yamashita, Keisuke Shindo, Norimitsu Kadowaki, Yoshio Koyanagi, Akifumi Takaori-Kondo

**Affiliations:** 1 Department of Hematology and Oncology, Graduate School of Medicine, Kyoto University, Kyoto, Japan; 2 Laboratory of Viral Pathogenesis, Institute for Virus Research, Kyoto University, Kyoto, Japan; University of Oslo, Norway

## Abstract

The transcription factor NF-κB plays a key regulatory role in lymphocyte activation and generation of immune response. Stimulation of T cell receptor (TCR) induces phosphorylation of CARMA1 by PKCθ, resulting in formation of CARMA1-Bcl10-MALT1 (CBM) complex at lipid rafts and subsequently leading to NF-κB activation. While many molecular events leading to NF-κB activation have been reported, it is less understood how this activation is negatively regulated. We performed a cell-based screening for negative regulators of TCR-mediated NF-κB activation, using mutagenesis and complementation cloning strategies. Here we show that casein kinase-2 interacting protein-1 (CKIP-1) suppresses PKCθ-CBM-NF-κB signaling. We found that CKIP-1 interacts with CARMA1 and competes with PKCθ for association. We further confirmed that a PH domain of CKIP-1 is required for association with CARMA1 and its inhibitory effect. CKIP-1 represses NF-κB activity in unstimulated cells, and inhibits NF-κB activation induced by stimulation with PMA or constitutively active PKCθ, but not by stimulation with TNFα. Interestingly, CKIP-1 does not inhibit NF-κB activation induced by CD3/CD28 costimulation, which caused dissociation of CKIP-1 from lipid rafts. These data suggest that CKIP-1 contributes maintenance of a resting state on NF-κB activity or prevents T cells from being activated by inadequate signaling. In conclusion, we demonstrate that CKIP-1 interacts with CARMA1 and has an inhibitory effect on PKCθ-CBM-NF-κB signaling.

## Introduction

The NF-κB family of transcription factors plays a key regulatory role in lymphocyte activation and generation of immune response [1]. The respective NF-κB target genes allow the organism to respond effectively to the environmental changes. Engagement of TCR by specific antigen presented on major histocompatibility complex (MHC) of antigen presenting cells (APC) induces T cell activation and proliferation. However, stimulation of TCR/CD3 complex alone is not sufficient for activation of NF-κB. The simultaneous costimulation of CD28 through its ligand, B7, is needed for optimal activation of NF-κB [2]. CD3/CD28 costimulation induces the formation of a large multicomponent complex at the contact site between T cell and the APC, termed as immunological synapse [3], [4]. This contact area of T cells is highly enriched in cholesterol and glycosphingo-lipids, also termed as lipid rafts, and serve as the platform for the assembly of proximal signaling components of TCR. PKCθ is recruited to the immunological synapse from the cytosol upon T cell stimulation and catalytically activated [5], [6]. Activated PKCθ phosphorylates CARMA1 (CARD11) to induce its conformational changes which enable CARMA1 to form the complex with Bcl10-MALT1 [7], [8]. Subsequently, the IκB kinase (IKK) complex becomes activated and phosphorylates IκBs, leading to their ubiquitylation and subsequent proteasomal degradation. The degradation of IκBs allows NF-κB to enter the nucleus and induce transcription of target genes [1].

CARMA1 is one of a family of caspase recruitment domain (CARD)- and membrane associated guanylate kinase-like (MAGUK) domain-containing proteins (CARMA) [9], [10]. CARMA1 contains an N-terminal CARD, followed by a coiled-coil (CC) domain, a PDZ domain, a Src homology 3 (SH3) domain, and a guanylate kinase (GUK)-like domain in the C-terminus. It has two mammalian homologs, CARMA2 and CARMA3. CARMA1 is predominantly expressed in spleen, thymus, and peripheral blood leukocyte (PBL); CARMA2 is expressed only in placenta; and CARMA3 is expressed in broad range of tissues but not in spleen, thymus or PBL. For B and T cells, the scaffold protein CARMA1 plays an essential role in antigen receptor-induced NF-κB activation [11]–[15]. Aberrant NF-κB activation could be involved in autoimmune diseases and malignant lymphomas. Constitutively active NF-κB in the activated B cell-like (ABC) subtype of diffuse large B cell lymphoma (DLBCL) can result from somatic mutations in genes involved in NF-κB signaling, such as CD79B, A20 and CARMA1 [16]. Recently, germline mutations in CARMA1 have also been reported in four patients with congenital B cell lymphocytosis [17]. Therefore CARMA1 activity needs to be tightly regulated.

Casein kinase-2 interacting protein-1 (CKIP-1) was originally identified as an interacting protein of casein kinase 2α (CK2α) [18]. CKIP-1 contains a pleckstrin homology (PH) domain at the N-terminus, a leucin zipper (LZ) motif at the C-terminus, and five proline-rich motifs throughout the protein [19]. Several interacting proteins of CKIP-1 have been identified and CKIP-1 plays scaffold roles in various signaling pathways [18]–[27]. It has also been reported that CKIP-1 binds to lipid through its PH domain and contributes to localization of its binding proteins. Genetically, CKIP-1-deficient mice show an age-dependent increase in bone mass as a result of accelerated osteogenesis, and the MEKK2-JNK-c-Jun/AP-1 axis is activated in CKIP-1 deficient mouse embryonic fibroblasts [22], [25]. However, the role of CKIP-1 in NF-κB signaling remains unknown.

Many findings leading to NF-κB activation have been reported, but it is less understood how this activation is negatively regulated. To elucidate negative regulation in TCR-mediated NF-κB activation, we have done a screening by mutagenesis and complementation cloning strategies. Here we report the identification of CKIP-1 as a negative regulator in NF-κB signaling via TCR. We show that CKIP-1 interacts with CARMA1, inhibits the interaction between PKCθ and CARMA1, and suppresses NF-κB activation.

## Materials and Methods

### Cells

CARMA1-deficient Jurkat T cell line, named JPM50.6, and JPM50.6/WT cell line, which was reconstituted with Myc-tagged CARMA1 wild type (WT) in JPM50.6, were kindly gifted from Dr. Xin Lin [11], [28]. These cell lines and Jurkat T cells were maintained with RPMI1640 (Nacalai Tesque, Kyoto, Japan) containing 10% fetal bovine serum (FBS) and 1% penicillin-streptomycin and glutamine (PSG) (Invitrogen, Carlsbad, USA). HEK293T cells were maintained with DMEM (Nacalai Tesque) containing 10% FBS and 1% PSG.

### Generation of mutant Jurkat T cells and complementation cloning strategies by lentiviral cDNA library

Jurkat T cell line stably expressing EGFP under the control of an NF-κB-dependent promoter, which we called JR-GFP, was kindly gifted from Dr. Xin Lin [11]. To generate mutant cells, JR-GFP cells were treated with 4 µg/ml of ICR191 (Sigma-Aldrich, St. Louis, USA), alkylating agent that typically generates random frame-shift mutations [11], [29], for 5 hr, and this treatment was repeated three times. After mutagenesis, EGFP-positive cells were sorted by BD FACSAria cell sorter (BD, New Jersey, USA) under the treatment with 2.5 µM of PKC inhibitor GF109203X (Sigma-Aldrich). Monoclonal mutant cell lines were derived by limiting dilution, and, among them, an NF-κB constitutively active cell line was identified. Human leukocyte cDNA library (Invitrogen) on pCS2-EF-GATEWAY-IRES-hrGFP was transferred into pCS2-EF-GATEWAY-IRES-H2K^k^ through LR reaction on Gateway cloning system (Invitrogen) and cDNA and H2K^k^-dual expressing lentiviral vector were prepared as described before [30], [31]. To identify NF-κB negative regulators, the NF-κB constitutively active cell line was infected with this viral vector. If the mutant phenotype was rescued by the gene from the library, EGFP expression might return to negative. Both H2K^k^-positive and EGFP-negative cells were sorted using BD FACSAria cell sorter and subjected to limiting dilution. If EGFP was normally induced by PMA/ionomycin in each single cell clone, the mutant phenotype should be rescued by the gene from the library. The genes rendering the reversion of the mutant phenotype were isolated by PCR using vector specific primers. Subsequent DNA sequencing and BLAST analysis should reveal the integrated gene.

### Plasmid constructs

Plasmids encoding Myc-CARMA1, Myc-CARMA1 truncated forms, EGFP-CARMA1, Myc-Bcl10, PKCθ WT, PKCθ AE [32], IKKβ, and GFP-NF-κB RelA were kind gifts from Dr. Xin Lin. Expression vectors for FLAG-CARMA1 and HA-Bcl10 were generated by subcloning of coding sequence into pcDNA3 vector (Invitrogen). GST-CARMA1 CD-CC was generated by subcloning of coding sequence into pGEX-4T-1 (GE Healthcare, Buckinghamshire, UK). Human CKIP-1 cDNA was generated by PCR amplification from Jurkat cDNA and cloned into pcDNA3/hygro and pcDNA3-FLAG vector (Invitrogen). Expression vector for DsRed-CKIP-1 was generated by subcloning of coding sequence into pDsRed1-N1 vector (Clontech, Mountain View, USA). ΔLZ-CKIP-1 and ΔPH-CKIP-1 truncated form were generated by PCR amplification from WT CKIP-1 expression vector and subcloned into pcDNA3/hygro vector.

### RNA interference

To identify a negative signaling component of NF-κB signaling from our candidates, we knocked down the molecules by specific siRNA in JR-GFP cells. siRNAs against our selected eighteen candidates were purchased from Thermo Scientific (Rockford, USA) (siGENOME SMARTpool). 5×10^6^ JR-GFP cells were electroporated with 400 pmol of non-targeting siRNA (D-001206-13), human TNFAIP3 (A20)-specific siRNA (M-009919-00), human CKIP-1-specific siRNA (M-016800-01), and siRNAs against other seventeen genes using AMAXA Nucleofector System (Lonza, Basel, Switzerland). Five days later, EGFP expression was analyzed by BD FACSCalibur. siRNA SMARTpool (Thermo Scientific) is a mixture of four siRNAs. We also used separate aliquot of four individual siRNAs (D-016800-01, 02, 03, 04).

### Chemicals, Cytokines, and Antibodies

PMA and ionomycin were purchased from Sigma-Aldrich. TNFα was from CellGenix (Freiburg, Germany). PE-conjugated anti-mouse H2K^k^ (CL9005PE) was from Cedarlane (Ontario, Canada). Anti-GFP (A6455) was from Molecular Probes (Eugene, USA). Mouse anti-human CD3 (555336), -CD28 (555725), and -PKCθ (610089) were from BD Biosciences (San Jose, USA). Anti-CKIP-1 (D-20, sc-50225), -IKKα/β (H470, sc-7607), and -Lck (3A5, sc-433) were from Santa Cruz Biotechnology (Santa Cruz, USA). Anti-β-actin (AC-15, A5441), -c-Myc (9E10, M5546, and C3956), and -FLAG (M2, F3165) were from Sigma-Aldrich. Anti-HA (12CA5) was from Roche (Mannheim, Germany). Anti-p-Erk (Thr202/Tyr204, E10, #9106), -Erk (#9102), and -CARMA1 (1D12, #4435) were from Cell Signaling Technology (Danvers, USA).

### Luciferase reporter assay

5 µg of 5xNF-κB-dependent luciferase (*Firefly*) reporter plasmid and 0.1 µg of EF1α promoter-dependent *Renilla* luciferase reporter were transfected together with 5 µg of plasmids encoding the desired genes or 400 pmol of siRNA by electroporation into 1×10^7^ Jurkat T cells in 0.4 ml serum-free RPMI1640 media at the power setting of 250 V and 950 µF. Nineteen hours later, the transfected cells were treated for 5 hr with plate-bound CD3 mAb (2 µg/ml), plate-bound CD3 + soluble CD28 mAb (2 µg/ml of each), TNFα (20 ng/ml), PMA (10 ng/ml), or PMA (10 ng/ml) + CD28 (2 µg/ml). NF-κB activity was measured with Dual-Luciferase Reporter Assay System (Promega, Madison, USA) and was determined by normalization of NF-κB-dependent *Firefly* luciferase to *Renilla* luciferase activity. Values represent the average of three independent experiments and error bars represent the SD from the average. Statistically significance was determined using Student's t test.

### Evaluation of NF-κB activity

Nuclear protein fractions were harvested by the Nuclear Extract kit (Active Motif, Carlsbad, USA). NF-κB activity was measured in 2 µg of nuclear protein extracts by the TransAM™ NF-κB p65 chemi (Active Motif), an ELISA-based kit to detect and quantify NF-κB p65 subunit activation. The assay was performed according to the manufacturer's protocol and analyzed using a microplate luminometer PerkinElmer 2030 ARVO™ X3 (PerkinElmer, Waltham, USA). Values represent the average of three independent experiments and error bars represent the SD from the average. Statistically significance was determined using Student's t test.

### Immunoprecipitation

For co-immunoprecipitation, 6-well plate HEK293T cells were transfected by the calcium phosphate method. Two days after transfection, cells were lysed in NP-40 lysis buffer (20 mM Tris-HCl pH 7.5, 250 mM NaCl, 1% NP-40) supplemented with 1 mM PMSF, protease inhibitor cocktail (Nacalai Tesque) and phosphatase inhibitor cocktail (Roche). Total cell lysates were precleared on Protein A Sepharose beads for 30 min at 4°C. The precleared cell lysates were immunoprecipitated with Protein A beads-conjugated with the desired antibodies for 6 hr. Immunoprecipitates were washed three times with lysis buffer.

To detect the protein interaction in JPM50.6/WT cells, 1×10^8^ cells were lysed in NP-40 lysis buffer (10 mM Tris-HCl pH 7.5, 150 mM NaCl, 0.5% NP-40) supplemented with protease inhibitor cocktail (Nacalai Tesque) and phosphatase inhibitor cocktail (Roche). Total cell lysates were precleared on Protein A Sepharose beads for 30 min at 4°C. The precleared cell lysates were immunoprecipitated with Protein A beads-conjugated with 2 µg of anti-CKIP-1 Ab at 4°C overnight. Immunoprecipitates were washed three times with 0.05% NP-40 buffer (10 mM Tris-HCl pH 7.5, 150 mM NaCl, 0.05% NP-40).

### Confocal microscopy

HEK293T cells were transfected with expression vectors and grown on the coverslips. 24 hr after transfection, the cells were incubated with Alexa Flour 488-conjugated cholera toxin B (Molecular probes) at 4°C for 20 min. The specimens were fixed with 4% paraformaldehyde in PBS and mounted on slides using ProLong Gold antifade reagent with DAPI (Invitrogen), and analyzed by confocal laser scanning fluorescence microscopy (Nikon Digital Eclipse C1).

### *In vitro* binding assay

FLAG-CKIP-1 was synthesized *in vitro* using the TNT T7 Quick Coupled Transcription/Translation System (Promega). GST and GST-CARMA1 CD-CC proteins were produced in *E. coli* BL21 and purified with glutathione Sepharose 4B beads (GE Healthcare). The beads were incubated with FLAG-CKIP-1 at 4°C for 2 hr. The beads were washed and proteins were eluted, followed by Western blotting with anti-FLAG antibody.

### Lipid raft purification

Costimulation of Jurkat T cells was performed in a final volume of 1 ml by addition of anti-CD3 (10 µg/ml) and anti-CD28 (5 µg/ml) antibodies, together with 15 µg of mouse IgG (Sigma-Aldrich). Cells (2×10^7^) were lysed in 1 ml MNE Buffer (25 mM MES pH 6.5, 150 mM NaCl, 5 mM EDTA) with 1% Triton-X, 1 mM PMSF, and protease inhibitor cocktail (Nacalai Tesque) for 20 min on ice and dounce homogenized 20 times. Samples were centrifuged at 1,000× g for 10 min at 4°C. The supernatants were mixed with 1 ml of OptiPrep (Axis-Shield, Oslo, Norway) and transferred to a Beckman Ultracentrifuge tube. Two milliliters of 30% OptiPrep followed by 1 ml of 5% OptiPrep in MNE buffer were overlaid. Samples were ultracentrifuged in a SW41Ti rotor (200,000× g for 20 hr). Fractions (400 µl per fraction) were collected from the top of the gradient. Proteins from each fraction were precipitated with trichloroacetic acid before separation by SDS-PAGE and Western blotting.

## Results

### Identification of CKIP-1 as a negative regulator of NF-κB activation

**Figure 1:**
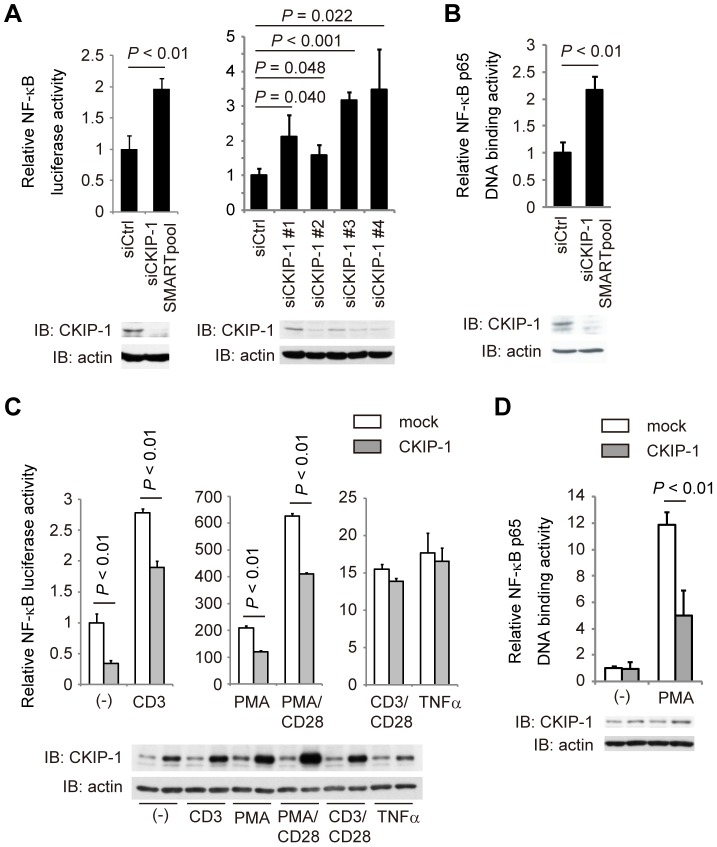
Identification of CKIP-1 as a negative regulator in NF-κB activation. (A) 400 pmol of human CKIP-1-specific siRNA or non-targeting siRNA together with 5 µg of κB-Luc, 0.1 µg of *Renilla*-Luc were electroporated into Jurkat T cells. Luciferase activity was assayed after 48 hr. The reduction of endogenous CKIP-1 protein levels was analyzed by Western blotting. (B) Jurkat T cells were electroporated with human CKIP-1-specific siRNA or non-targeting siRNA using AMAXA Nucleofector System (Lonza). Thirty hours later, nuclear protein extracts were harvested and NF-κB activity was measured by TransAM NF-κB p65 chemi kit (Active Motif). The reduction of endogenous CKIP-1 protein levels was analyzed by Western blotting. (C) Jurkat T cells were transfected with 5 µg of CKIP-1 or empty vector (mock) together with 5 µg of κB-Luc and 0.1 µg of *Renilla*-Luc. Nineteen hours later, cells were stimulated for 5 hr upon CD3 (2 µg/ml), CD3/CD28 (2 µg/ml each), TNFα (20 ng/ml), PMA (10 ng/ml) or PMA (10 ng/ml) + CD28 (2 µg/ml). The expressed protein levels were analyzed by Western blotting. (D) Jurkat T cells were transfected with 5 µg of CKIP-1 or empty vector (mock). Twenty-four hours later, cells were stimulated for 30 min upon PMA (10 ng/ml). Then cells were harvested and NF-κB activity was measured by TransAM NF-κB p65 chemi kit. The expressed protein levels were analyzed by Western blotting. Values represent the average of three independent experiments and error bars represent the SD from the average.

We have performed a cell-based screening to find negative regulators in TCR-mediated NF-κB activation, using somatic mutagenesis and complementation cloning strategies [11], [29]. We used Jurkat T cell line expressing EGFP under the control of an NF-κB-dependent promoter, named JR-GFP [11]. To generate NF-κB constitutively active cell lines, JR-GFP cells were subjected to mutagenesis with ICR191, and EGFP-positive cells were sorted under the treatment of PKC inhibitor GF109203X. After limiting dilution, we identified an NF-κB constitutively active cell line in which negative regulators for NF-κB activation must be mutated. To identify NF-κB negative regulators, the NF-κB constitutively active cell line was infected with a human leukocyte-cDNA library expressing lentivirus, and EGFP-negative cells were sorted. If the mutant phenotype was rescued by transduction of the gene from the library, EGFP expression would return to negative. The genes rendering the reversion of the mutant phenotype were isolated by PCR and sequenced using library vector specific primers, and then we obtained dozens of candidates for NF-κB negative regulators. To examine whether any of these candidates downregulate NF-κB activity, we selected and knocked down eighteen molecules by specific siRNA in JR-GFP cells. We found that knockdown of CKIP-1 induced expression of EGFP more than that of TNFAIP3 (A20), which was known as a negative regulator of NF-κB and used as a positive control [33] (Figure S1). To confirm that CKIP-1 was a negative regulator of NF-κB, Jurkat T cells were transfected with CKIP-1 siRNA together with an NF-κB-dependent luciferase reporter plasmid. We used siRNA SMARTpool, which is a mixture of four siRNAs, and separate aliquot of all four individual siRNAs. Knockdown of CKIP-1 increased NF-κB activity (Figure 1A). We also showed that knockdown of CKIP-1 induced DNA binding activity of NF-κB p65 (Figure 1B), by using the transcription factor DNA-binding ELISA. Thus, we clearly demonstrated that CKIP-1 was a novel NF-κB negative regulator.

### CKIP-1 suppresses NF-κB activation induced by PMA and constitutively active PKCθ

**Figure 2:**
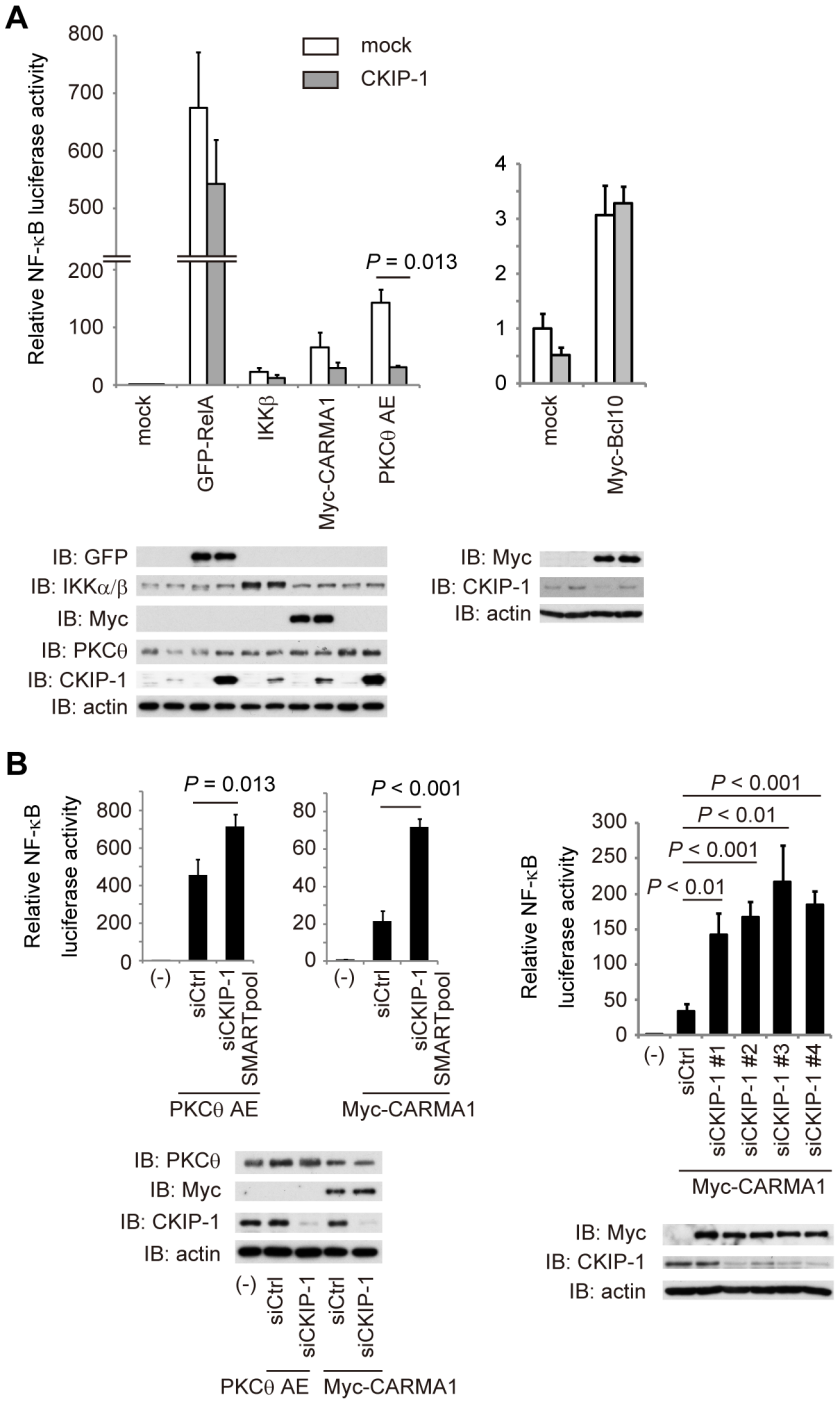
CKIP-1 suppresses NF-κB activation induced by constitutively active PKCθ. (A) Jurkat T cells were transfected with 5 µg of CKIP-1 or empty vector (mock) together with 5 µg of each signaling component, 5 µg of κB-Luc and 0.1 µg of *Renilla*-Luc by electroporation. Luciferase activity was assayed after 24 hr. PKCθ AE is constitutively active mutant. The expressed protein levels were analyzed by Western blotting. (B) Jurkat T cells were transfected with 400 pmol of human CKIP-1-specific siRNA or non-targeting siRNA together with 5 µg of κB-Luc, 0.1 µg of *Renilla*-Luc, and 5 µg of PKCθ AE (left panel) or CARMA1 (right panel) by electroporation. Thirty hours later, cells were lysed and luciferase activity was assayed. The expressed protein levels were analyzed by Western blotting. Values represent the average of three independent experiments and error bars represent the SD from the average.

To examine whether the downregulation of NF-κB activation by CKIP-1 is specific to TCR stimulation, Jurkat T cells transfected with CKIP-1, treated with different stimulation, and assessed NF-κB activity by luciferase reporter assays. CKIP-1 suppressed NF-κB activity in unstimulated cells and stimulated by CD3, PMA and PMA/CD28, but not by TNFα or CD3/CD28 (Figure 1C). Using the transcription factor DNA-binding ELISA, we also showed that CKIP-1 suppressed NF-κB activation induced by PMA stimulation (Figure 1D). These data suggest that CKIP-1 inhibits NF-κB signaling via TCR but not via TNF receptor and that CKIP-1 targets downstream signaling components of PKCθ, since the treatment of PMA directly activates PKCs. To clarify which step of signaling CKIP-1 affects, NF-κB activation driven by transfection of each downstream signaling component of PKCθ was assessed in Jurkat T cells in the presence or absence of co-transfection of CKIP-1 (Figure 2A). NF-κB activation induced by PKCθ AE, a constitutively active mutant [32], was clearly suppressed by CKIP-1, whereas activation induced by NF-κB RelA, IKKβ or Bcl10 was not affected. NF-κB activation induced by CARMA1 seemed to be suppressed by CKIP-1, but the effect was not statistically significant. Conversely, knockdown of CKIP-1 increased NF-κB activation induced by transfection of CARMA1 or PKCθ AE (Figure 2B). These results suggest that the inhibitory effect of CKIP-1 targets signaling events around PKCθ or CARMA1.

**Figure 3:**
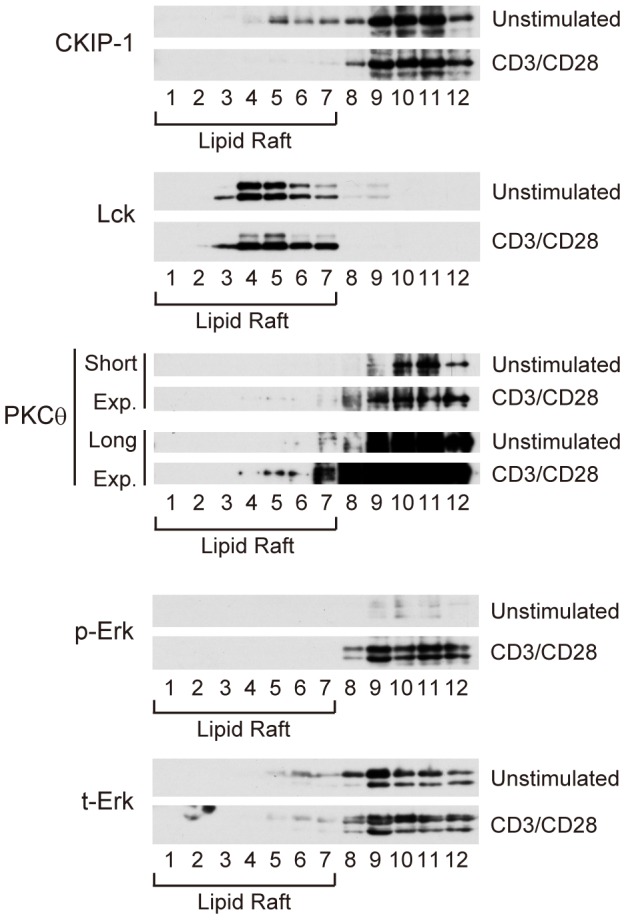
Lipid rafts accumulated by CD3/CD28 costimulation do not contain CKIP-1. Jurkat T cells were stimulated for 15-CD3 (10 µg/ml) and anti-CD28 (5 µg/ml), together with 15 µg of mouse IgG. The cells were then lysed and subjected to OptiPrep density gradient centrifugation to isolate lipid rafts. Lysates were subjected to SDS-PAGE and analyzed by Western blotting.

As shown in Figure 1C, CKIP-1 did not suppress CD3/CD28-induced NF-κB activation. We hypothesized that CKIP-1 might work in a resting state and finish its role during CD3/CD28 costimulation. PKCθ and CARMA1 have been reported to be recruited to lipid rafts upon TCR stimulation [34]. It has been shown that CKIP-1 binds to lipid through its PH domain and overexpressed CKIP-1 localizes in the plasma membrane and partly in the nucleus [18], [20], [22]. To examine where CKIP-1 localizes in Jurkat T cells, the detergent-insoluble membrane (lipid raft) fractions were prepared by the ultra-centrifugation in a discontinuous OptiPrep density gradient. Lck was constitutively associated with lipid rafts, and PKCθ was recruited to lipid rafts after CD3/CD28 costimulation (Figure 3) as previously reported [28], [35]. Phosphorylation of Erk was induced by CD3/CD28 costimulation. CKIP-1 partly localized at lipid rafts in unstimulated Jurkat T cells, and intriguingly, CKIP-1 was excluded from lipid rafts when cells were stimulated upon CD3/CD28 (Figure 3). These data suggest that, when cells are stimulated upon CD3/CD28 and lipid rafts are accumulated, CKIP-1 localizes out of the lipid rafts and its inhibitory effect does not extend.

### Identification of CARMA1 as a binding partner of CKIP-1

**Figure 4:**
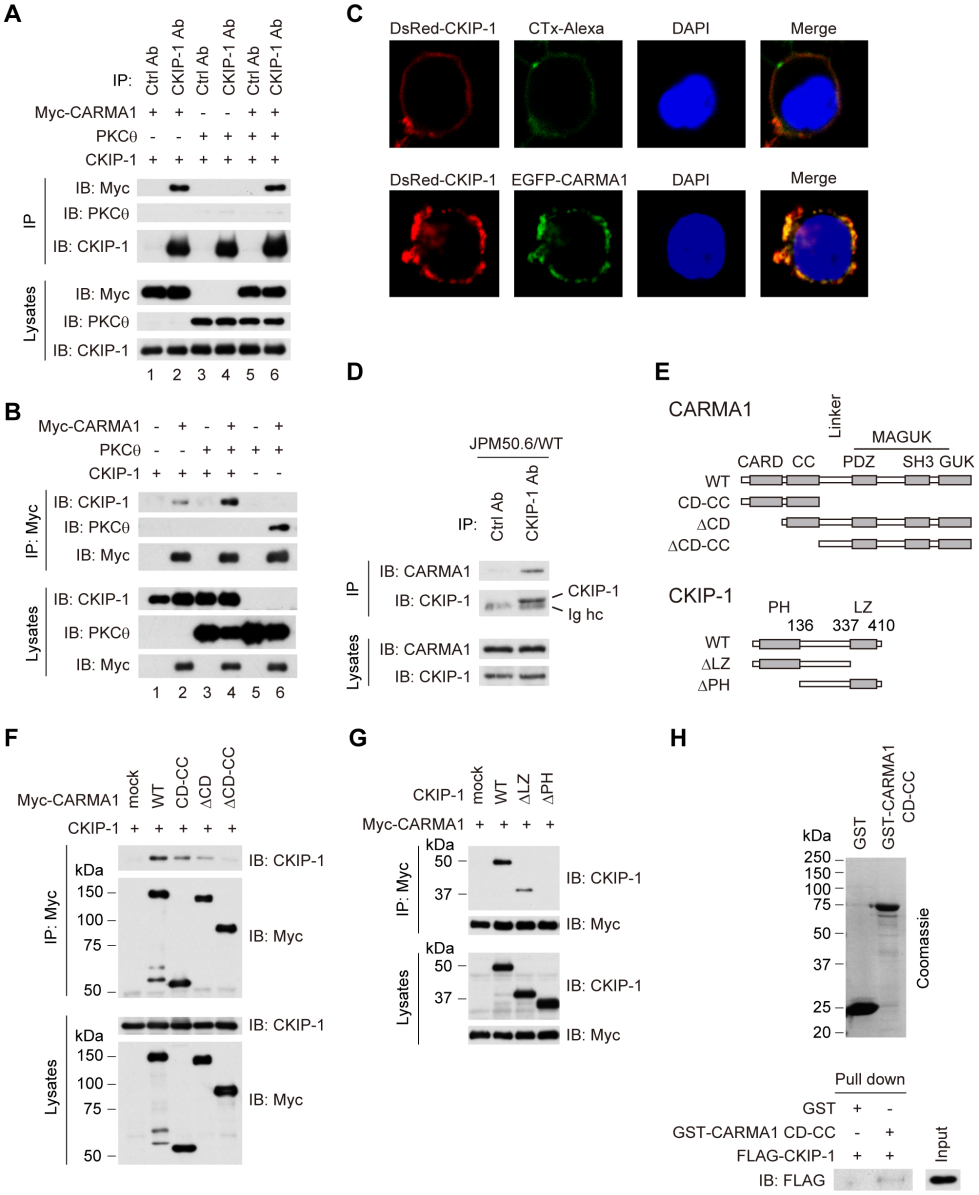
Identification of CARMA1 as a binding partner of CKIP-1. (A) HEK293T cells were transfected with plasmids encoding CKIP-1 together with Myc-CARMA1 or PKCθ, lysed, and immunoprecipitated by anti-CKIP-1 or control antibody. (B) HEK293T cells were co-transfected with plasmids encoding Myc-CARMA1, PKCθ or CKIP-1, lysed, and immunoprecipitated by anti-Myc antibodies. (C) HEK293T cells were transfected with DsRed-CKIP-1. 24 hr later, the transfected cells were incubated with Alexa Flour 488-conjugated cholera toxin B (CTx), and were fixed and stained with DAPI. In lower panels, HEK293T cells were transfected with DsRed-CKIP-1 together with EGFP-CARMA1. 24 hr later, cells were fixed and stained with DAPI. The localization of CKIP-1, CARMA1 and Alexa Flour 488-CTx-labeled lipid rafts was visualized by confocal microscopy. (D) JPM50.6/WT cells, which were reconstituted by Myc-CARMA1 WT into CARMA1-deficient Jurkat T cells, were lysed and immunoprecipitated by anti-CKIP-1 or control antibody. Ig hc, immunoglobulin heavy chain. (E) Schematic diagram of CARMA1 and CKIP-1 truncated forms used in the experiments. CARD, caspase recruitment domain; CC, coiled-coil; SH3, Src homology 3; GUK, guanylate kinase; MAGUK, membrane-associated GUK; PH, pleckstrin homology; LZ, leucin zipper. (F) HEK293T cells were transfected with CKIP-1 together with Myc-CARMA1 truncated form, lysed, and immunoprecipitated by anti-Myc antibody, followed by Western blotting with indicated antibodies. (G) HEK293T cells were transfected with Myc-CARMA1 together with each CKIP-1 truncated form. Cell lysates were immunoprecipitated by anti-Myc antibody, followed by Western blotting with indicated antibodies. (H) CARMA1 CD-CC was purified from *E. coli* as a GST fusion protein. GST alone or GST-tagged CARMA1 CD-CC was incubated with *in vitro* transcribed/translated FLAG-CKIP-1. GST pull-downs and input were subjected to Western blotting with anti-FLAG antibody.

To determine the interacting partner of CKIP-1, we examined whether CKIP-1 associates with PKCθ or CARMA1 by co-immunoprecipitation assays in HEK293T cells. CKIP-1 interacted with Myc-CARMA1 but not with PKCθ (Figure 4A, lane 1–4, Figure 4B, lane 1 and 2). In the presence of co-transfection of PKCθ, CKIP-1 also interacts with Myc-CARMA1 (Figure 4A, lane 5 and 6, Figure 4B, lane 3 and 4). We examined CKIP-1 and CARMA1 localization in HEK293T cells by confocal microscopy. HEK293T cells were transfected with DsRed-CKIP-1 and EGFP-CARMA1. The lipid rafts of transfected cells were stained with Alexa Flour 488-conjugated cholera toxin B (CTx). DsRed-CKIP-1 colocalized with Alexa Flour 488-CTx-labeled lipid rafts (Figure 4C, upper panel), and colocalized extensively with EGFP-CARMA1 (Figure 4C, lower panel). This result indicates that CKIP-1 colocalizes with CARMA1 at the plasma membrane. Next, we examined the interaction between CKIP-1 and CARMA1 in T cells, using JPM50.6/WT cells, which were reconstituted with Myc-CARMA1 wild type (WT) in CARMA1-deficient Jurkat (JPM50.6) T cells [11], [28]. Myc-CARMA1 was co-immunoprecipitated with endogenous CKIP-1 but not with control IgG (Figure 4D). To determine the domain of CARMA1 that was critical for the interaction with CKIP-1, truncated forms of CARMA1 were tested (Figure 4E). Co-immunoprecipitation assays showed that CKIP-1 bound to CARMA1 WT, CD-CC and ΔCD, but not to ΔCD-CC (Figure 4F), indicating that CKIP-1 associates with the CC domain of CARMA1. To determine the responsible region in CKIP-1 for the association with CARMA1, we generated several CKIP-1 truncated forms (Figure 4E). Co-immunoprecipitation assays revealed that CKIP-1 WT and ΔLZ bound to CARMA1 but CKIP-1 ΔPH did not (Figure 4G), indicating that the PH domain of CKIP-1 was essential for the interaction with CARMA1. To investigate direct interaction between CKIP-1 and CARMA1, *in vitro* GST pull-down assay was performed. GST-tagged CARMA1 CD-CC was able to interact with FLAG-CKIP-1 but GST was not (Figure 4H). Together, CARMA1 is a specific interacting partner of CKIP-1.

### PH domain of CKIP-1 is essential for the interaction with CARMA1 and the inhibitory effect on NF-κB activation

**Figure 5:**
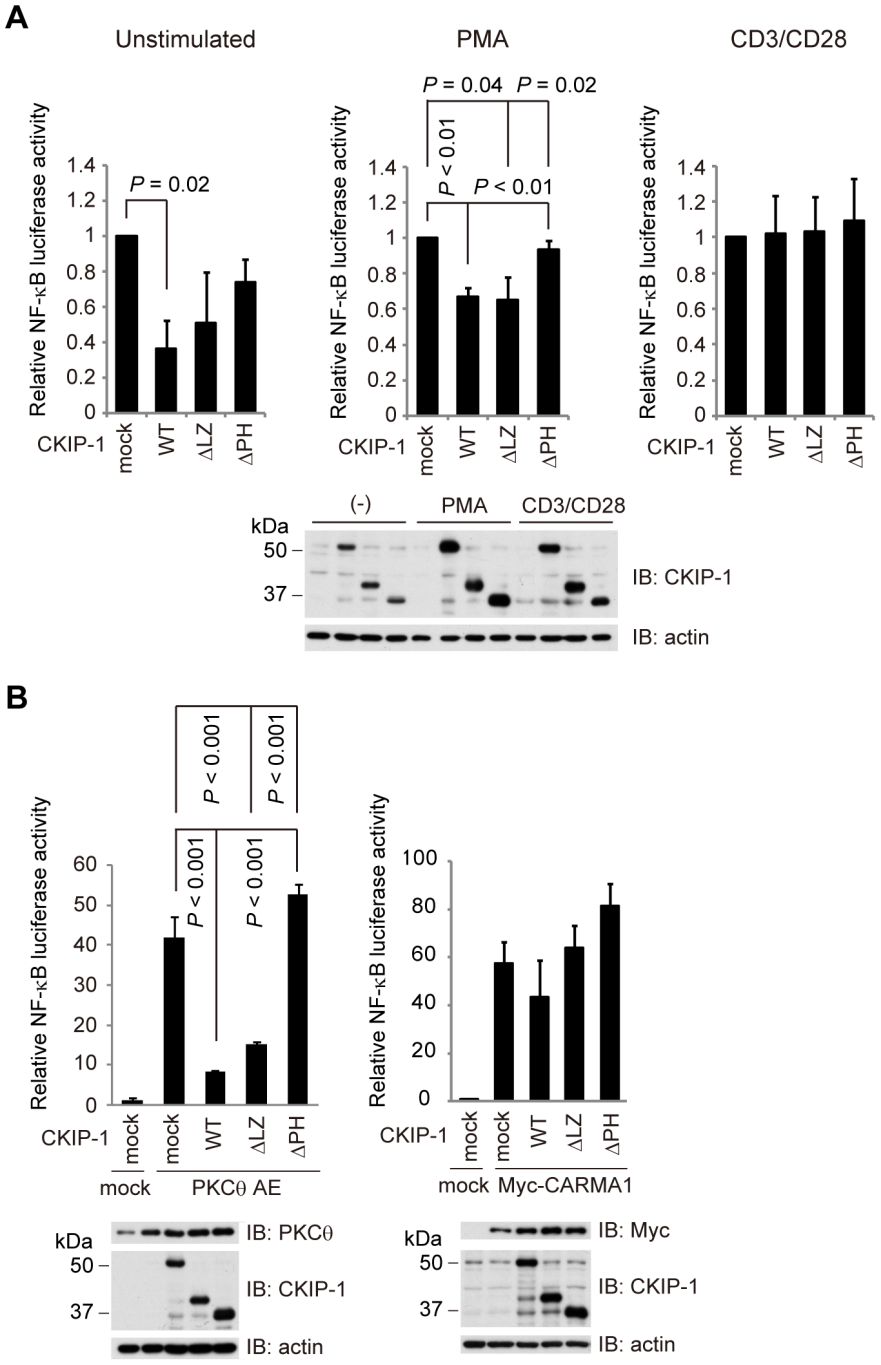
PH domain of CKIP-1 is essential not only for the interaction with CARMA1 but also for the inhibitory effect on NF-κB activation. (A) Jurkat T cells were electroporated with 5 µg of each CKIP-1 truncated form together with 5 µg of κB-Luc and 0.1 µg of *Renilla*-Luc. Nineteen hours later, cells were stimulated for 5 hr upon PMA (10 ng/ml) or CD3/CD28 (2 µg/ml each). The expressed protein levels were analyzed by Western blotting. (B) Jurkat T cells were electroporated with 5 µg of each CKIP-1 truncated form together with 5 µg of PKCθ AE or Myc-CARMA1, 5 µg of κB-Luc and 0.1 µg of *Renilla*-Luc. After 24 hr, cells were lysed and luciferase activity was assessed. The expressed protein levels were analyzed by Western blotting. Values represent the average of three independent experiments and error bars represent the SD from the average.

Next we examined the function of each truncated form of CKIP-1 on NF-κB activation, using luciferase reporter assays. Jurkat T cells were transfected with each CKIP-1 truncated form and stimulated by PMA and CD3/CD28. CKIP-1 WT and ΔLZ inhibited NF-κB activation induced by stimulation with PMA, but CKIP-1 ΔPH did not (Figure 5A, middle panel). Similarly to CKIP-1 WT (Figure 1C), the truncated forms of CKIP-1 gave no influence upon CD3/CD28-induced-NF-κB activation (Figure 5A, right panel). In resting state, the effect of the truncated forms was not statistically significant, because of the little amount of NF-κB activity in unstimulated cells (Figure 5A, left panel). Jurkat T cells were transfected with PKCθ AE together with each CKIP-1 truncated form. CKIP-1 WT and ΔLZ suppressed NF-κB activation, but CKIP-1 ΔPH did not (Figure 5B, left panel). As shown in Figure 2A, NF-κB activation induced by CARMA1 seemed to be suppressed by CKIP-1 WT, but the effect was not statistically significant. Neither CKIP-1 ΔLZ nor ΔPH repressed NF-κB activation induced by CARMA1 (Figure 5B, right panel). These results suggest that PH domain of CKIP-1, which is required for association with CARMA1, is essential for the inhibitory effect on NF-κB activation.

### CKIP-1 inhibits the interaction between PKCθ and CARMA1

**Figure 6:**
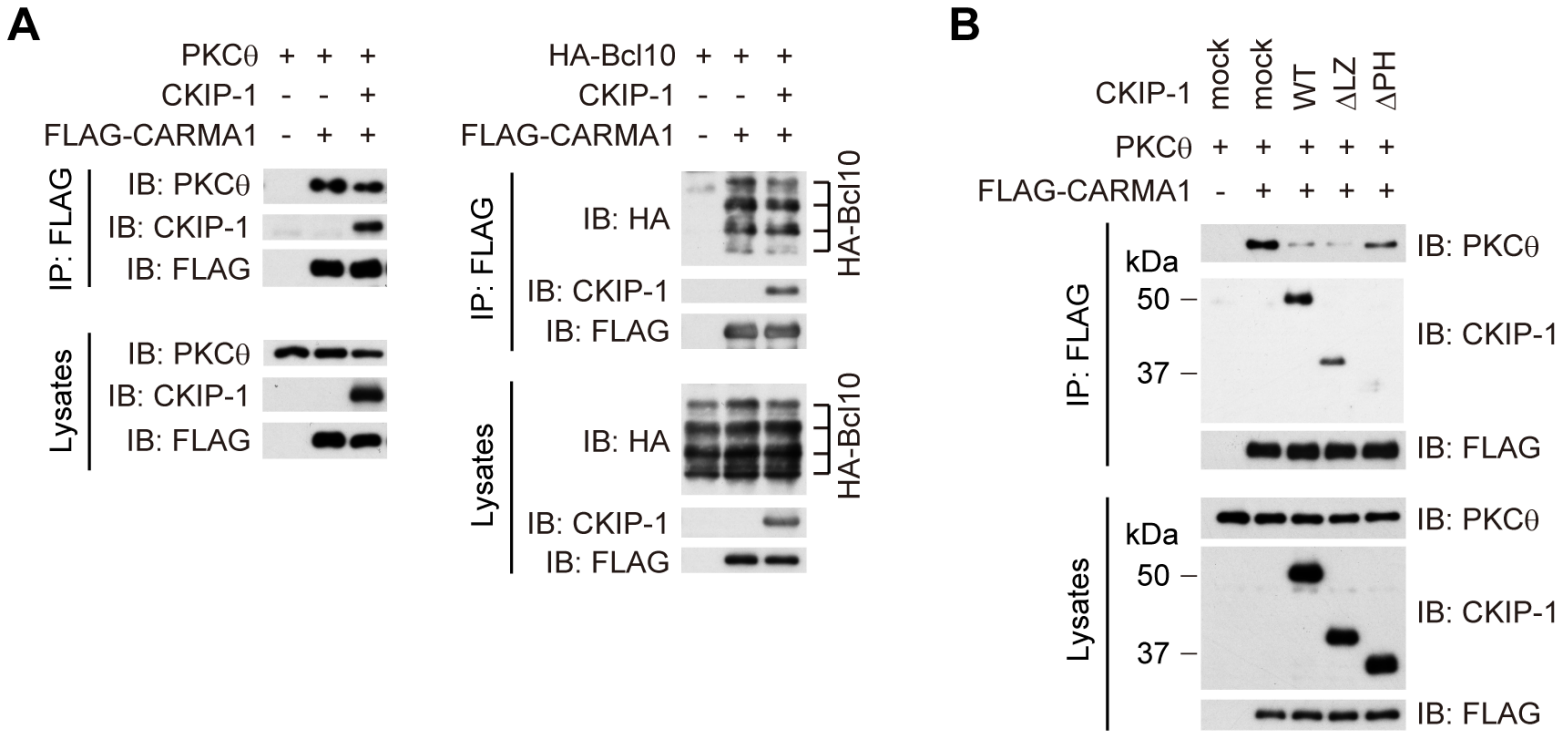
CKIP-1 inhibits the interaction between PKCθ and CARMA1. (A) HEK293T cells were transfected with CKIP-1 or empty vector (mock) together with PKCθ and FLAG-CARMA1 (left panel), or together with HA-Bcl10 and FLAG-CARMA1 (right panel). Cell lysates were immunoprecipitated by anti-FLAG antibody, followed by Western blotting with indicated antibodies. (B) HEK293T cells were transfected with CKIP-1 truncated form together with PKCθ and FLAG-CARMA1. Cell lysates were immunoprecipitated by anti-FLAG antibody, followed by Western blotting with indicated antibodies.

PKCθ phosphorylates CARMA1 in its Linker between the CD-CC domain and the MAGUK domain, which induces conformational change of CARMA1 [7], [8]. Then CARMA1 binds to Bcl10 through CARD-CARD interaction [9], [36]. Since our data suggested that CKIP-1 interacted with the CC domain of CARMA1, we hypothesized that CKIP-1 might inhibit the interaction between CARMA1 and PKCθ or between CARMA1 and Bcl10. Co-immunoprecipitation assays showed that CKIP-1 inhibited the interaction between PKCθ and CARMA1, but not that between CARMA1 and Bcl10 (Figure 6A). As shown in Figure 4B (lane 5 and 6), PKCθ was immunoprecipitated with Myc-CARMA1, but, in the presence of co-transfection of CKIP-1, the interaction between PKCθ and CARMA1 was diminished (Figure 4B, lane 3 and 4). Next, we examined the inhibitory effect of CKIP-1 truncated forms on the interaction between PKCθ and CARMA1. Consistent with the results of the binding and the inhibitory effect of the truncated forms (Figure 4G and Figure 5), CKIP-1 WT and ΔLZ inhibited the interaction between PKCθ and CARMA1, although CKIP-1 ΔPH showed no effect (Figure 6B). These results suggest that CKIP-1 suppresses NF-κB activation by inhibiting the interaction between PKCθ and CARMA1.

## Discussion

NF-κB signaling in antigen-stimulated lymphocytes plays an important role in immune response. Aberrant NF-κB activation has been shown to be involved in autoimmune diseases and malignant lymphomas. Especially, altered expression and/or function of CBM proteins have been reported in the ABC subtype of DLBCL [16], [37], [38] and MALT lymphoma [39].

In this study, we show that CKIP-1 is a novel interacting protein with CARMA1 and acts as a suppressor of NF-κB signaling. Our results suggest that CKIP-1 suppresses NF-κB signaling by inhibiting the interaction between PKCθ and CARMA1. However, CKIP-1 does not suppress NF-κB activation induced by CD3/CD28 costimulation. Our data suggest that it is because CKIP-1 localizes outside of the lipid rafts and its inhibitory effect does not extend, when cells are stimulated upon CD3/CD28 and lipid rafts are accumulated. A transmembrane adaptor molecule PAG/Cbp is also a negative regulator of T cell activation. In resting T cells, PAG/Cbp is phosphorylated by Lck and interacts with C-terminal Src kinase (Csk), which inhibits T cell activation by suppressing c-Src. In response to stimulation of TCR, PAG/Cbp becomes rapidly dephosphorylated and dissociates from Csk [40], [41]. Likewise, IκBs usually retain NF-κB in the cytoplasm through physical interaction. In response to signaling, IκBs are phosphorylated, leading to their ubiquitylation and subsequent proteasomal degradation [42]. Similarly to PAG/Cbp or IκBs, CKIP-1 usually interacts with CARMA1, but its inhibitory effect might be abrogated during CD3/CD28 costimulation. We presume that CKIP-1 physiologically prevents T cells from being activated by inadequate stimulation and might play a role like a gatekeeper for correct CD3/CD28 signaling at the step of CARMA1 during antigen-stimulation. We speculate that, in resting T cells, CKIP-1 associates with CARMA1 and keeps PKCθ away from CARMA1. Our date clearly showed that when T cells are stimulated appropriately upon CD3/CD28 costimulation, both PKCθ and CARMA1 are recruited to lipid rafts. However, CKIP-1 remains outside of the lipid rafts, and its inhibitory effect cannot extend. CARMA1 is then phosphorylated by PKCθ at the lipid rafts leading to its conformational change into an active form. The activated CARMA1 recruits Bcl10-MALT1 complex and subsequently induces NF-κB activation.

PAG/Cbp-deficient mice exhibit no overt phenotype [43], [44], but, in cancer cells, PAG/Cbp is involved in repressing the oncogenecity of c-Src [45]. CKIP-1-deficient mice are reported to undergo an age-dependent increase in bone mass [25]. However, no phenotype about immune disorders or neoplasm has been described. Thus, PAG/Cbp and CKIP-1 might be dispensable or could be compensated by some other negative regulators, because multiple checkpoints through TCR-mediated NF-κB signaling are likely to be independently required to prevent the unwarranted expansion and transformation of lymphocytes, and to ensure an appropriate adaptive immune response. Our data suggest that the suppression of CKIP-1 can work in a resting state or against aberrant PKCθ activation such as expression of constitutively active PKCθ or treatment of PMA. Similarly to PAG/Cbp, only in malignant lymphomas or immunological disorders, CKIP-1 might play a critical role as a suppressor of aberrant NF-κB activation.

Recently, novel germline CARMA1 mutations have been reported in four patients with congenital B cell lymphocytosis [17]. These CARMA1 mutants constitutively drive NF-κB activation, resulting in elevated NF-κB activity and increased proliferation of patient primary B cells. However, patient primary T cells expressing these CARMA1 mutants are hyporesponsive to CD3/CD28 costimulation. It has also been reported that chronic NF-κB activation, triggered by transgenic expression of constitutively active IKKβ in mice, renders T cells hyporesponsive to TCR stimulation [46]. We speculate that T cells have the mechanism by which an anergic state is induced by chronic active NF-κB signaling, and it might be one of the reasons why knockdown of CKIP-1 did not exhibit clear phenotypes in TCR stimulation. Analysis of B cells might be useful for deciphering the physiological role of CKIP-1.

There have been already reported two inhibitory regulators that interact with CARMA1. The kinesin GAKIN negatively regulates occupancy of CARMA1 at the center of the immunological synapse, and limits the extent of signaling [47]. Casein kinase 1α (CK1α), which is reported to be a bifunctional regulator, also interacts with CARMA1 and terminates signaling by phosphorylating CARMA1 [48]. Although CKIP-1 interacts with CARMA1 as GAKIN and CK1α do, CKIP-1 shows several different aspects. Whereas GAKIN competes with Bcl10 for binding, CKIP-1 competes with PKCθ but not with Bcl10. GAKIN and CK1α associate with CARMA1 in a signal-dependent manner. On the other hand, CKIP-1 neither localizes at lipid rafts nor influences NF-κB activation during CD3/CD28 costimulation. To our knowledge, CKIP-1 is the first molecule that negatively regulates CARMA1 in a resting state or in aberrantly activated signaling.

In conclusion, we have herein demonstrated an inhibitory effect of CKIP-1 in PKCθ-CBM-NF-κB signaling. CKIP-1 interacts with CARMA1 and competes with PKCθ for binding. It suggests that CKIP-1 plays a unique role to keep resting T cells in a quiescent state or to prevent T cells from being activated by inadequate signaling. Dysfunction of CKIP-1 might constitutively activate NF-κB, leading to autoimmune diseases or malignant lymphomas, and the signaling events around CKIP-1 might be good therapeutic targets.

## Supporting Information

Figure S1 **Knockdown of CKIP-1 induces NF-κB activation.** The JR-GFP cells were electroporated with 400 pmol of non-targeting siRNA, or specific siRNA against each gene by AMAXA Nucleofector System. Five days later, the expression of EGFP was assessed by FACS.
(TIF)

## References

[1] Vallabhapurapu S, Karin M (2009) Regulation and function of NF-kappaB transcription factors in the immune system. Annu Rev Immunol 27: 693–733. 19302050 10.1146/annurev.immunol.021908.132641

[2] Kane LP, Lin J, Weiss A (2002) It's all Rel-ative: NF-kappaB and CD28 costimulation of T-cell activation. Trends Immunol 23: 413–420. 12133805 10.1016/s1471-4906(02)02264-0

[3] Dustin ML, Olszowy MW, Holdorf AD, Li J, Bromley S, et al (1998) A novel adaptor protein orchestrates receptor patterning and cytoskeletal polarity in T-cell contacts. Cell 94: 667–677. 9741631 10.1016/s0092-8674(00)81608-6

[4] Dustin ML (2008) T-cell activation through immunological synapses and kinapses. Immunol Rev 221: 77–89. 18275476 10.1111/j.1600-065X.2008.00589.x

[5] Chuang HC, Lan JL, Chen DY, Yang CY, Chen YM, et al (2011) The kinase GLK controls autoimmunity and NF-kappaB signaling by activating the kinase PKC-theta in T cells. Nat Immunol 12: 1113–1118. 21983831 10.1038/ni.2121

[6] Kong KF, Yokosuka T, Canonigo-Balancio AJ, Isakov N, Saito T, et al (2011) A motif in the V3 domain of the kinase PKC-theta determines its localization in the immunological synapse and functions in T cells via association with CD28. Nat Immunol 12: 1105–1112. 21964608 10.1038/ni.2120 PMC3197934

[7] Matsumoto R, Wang D, Blonska M, Li H, Kobayashi M, et al (2005) Phosphorylation of CARMA1 plays a critical role in T Cell receptor-mediated NF-kappaB activation. Immunity 23: 575–585. 16356856 10.1016/j.immuni.2005.10.007

[8] Sommer K, Guo B, Pomerantz JL, Bandaranayake AD, Moreno-Garcia ME, et al (2005) Phosphorylation of the CARMA1 linker controls NF-kappaB activation. Immunity 23: 561–574. 16356855 10.1016/j.immuni.2005.09.014

[9] Thome M, Charton JE, Pelzer C, Hailfinger S (2010) Antigen receptor signaling to NF-kappaB via CARMA1, BCL10, and MALT1. Cold Spring Harb Perspect Biol 2: a003004. 20685844 10.1101/cshperspect.a003004 PMC2926749

[10] Blonska M, Lin X (2011) NF-kappaB signaling pathways regulated by CARMA family of scaffold proteins. Cell Res 21: 55–70. 21187856 10.1038/cr.2010.182 PMC3193407

[11] Wang D, You Y, Case SM, McAllister-Lucas LM, Wang L, et al (2002) A requirement for CARMA1 in TCR-induced NF-kappa B activation. Nat Immunol 3: 830–835. 12154356 10.1038/ni824

[12] Egawa T, Albrecht B, Favier B, Sunshine MJ, Mirchandani K, et al (2003) Requirement for CARMA1 in antigen receptor-induced NF-kappa B activation and lymphocyte proliferation. Curr Biol 13: 1252–1258. 12867038 10.1016/s0960-9822(03)00491-3

[13] Hara H, Wada T, Bakal C, Kozieradzki I, Suzuki S, et al (2003) The MAGUK family protein CARD11 is essential for lymphocyte activation. Immunity 18: 763–775. 12818158 10.1016/s1074-7613(03)00148-1

[14] Jun JE, Wilson LE, Vinuesa CG, Lesage S, Blery M, et al (2003) Identifying the MAGUK protein Carma-1 as a central regulator of humoral immune responses and atopy by genome-wide mouse mutagenesis. Immunity 18: 751–762. 12818157 10.1016/s1074-7613(03)00141-9

[15] Newton K, Dixit VM (2003) Mice lacking the CARD of CARMA1 exhibit defective B lymphocyte development and impaired proliferation of their B and T lymphocytes. Curr Biol 13: 1247–1251. 12867037 10.1016/s0960-9822(03)00458-5

[16] Shaffer AL 3rd, Young RM, Staudt LM (2012) Pathogenesis of human B cell lymphomas. Annu Rev Immunol 30: 565–610. 22224767 10.1146/annurev-immunol-020711-075027 PMC7478144

[17] Snow AL, Xiao W, Stinson JR, Lu W, Chaigne-Delalande B, et al (2012) Congenital B cell lymphocytosis explained by novel germline CARD11 mutations. J Exp Med 209: 2247–2261. 23129749 10.1084/jem.20120831 PMC3501355

[18] Bosc DG, Graham KC, Saulnier RB, Zhang C, Prober D, et al (2000) Identification and characterization of CKIP-1, a novel pleckstrin homology domain-containing protein that interacts with protein kinase CK2. J Biol Chem 275: 14295–14306. 10799509 10.1074/jbc.275.19.14295

[19] Nie J, Liu L, He F, Fu X, Han W, et al (2013) CKIP-1: a scaffold protein and potential therapeutic target integrating multiple signaling pathways and physiological functions. Ageing Res Rev 12: 276–281. 22878216 10.1016/j.arr.2012.07.002

[20] Safi A, Vandromme M, Caussanel S, Valdacci L, Baas D, et al (2004) Role for the Pleckstrin Homology Domain-Containing Protein CKIP-1 in Phosphatidylinositol 3-Kinase-Regulated Muscle Differentiation. Mol Cell Biol 24: 1245–1255. 14729969 10.1128/MCB.24.3.1245-1255.2004 PMC321442

[21] Canton DA, Olsten ME, Kim K, Doherty-Kirby A, Lajoie G, et al (2005) The pleckstrin homology domain-containing protein CKIP-1 is involved in regulation of cell morphology and the actin cytoskeleton and interaction with actin capping protein. Mol Cell Biol 25: 3519–3534. 15831458 10.1128/MCB.25.9.3519-3534.2005 PMC1084316

[22] Zhang L, Xing G, Tie Y, Tang Y, Tian C, et al (2005) Role for the pleckstrin homology domain-containing protein CKIP-1 in AP-1 regulation and apoptosis. EMBO J 24: 766–778. 15706351 10.1038/sj.emboj.7600532 PMC549613

[23] Zhang L, Tie Y, Tian C, Xing G, Song Y, et al (2006) CKIP-1 recruits nuclear ATM partially to the plasma membrane through interaction with ATM. Cell Signal 18: 1386–1395. 16325375 10.1016/j.cellsig.2005.10.017

[24] Tokuda E, Fujita N, Oh-hara T, Sato S, Kurata A, et al (2007) Casein kinase 2-interacting protein-1, a novel Akt pleckstrin homology domain-interacting protein, down-regulates PI3K/Akt signaling and suppresses tumor growth in vivo. Cancer Res 67: 9666–9676. 17942896 10.1158/0008-5472.CAN-07-1050

[25] Lu K, Yin X, Weng T, Xi S, Li L, et al (2008) Targeting WW domains linker of HECT-type ubiquitin ligase Smurf1 for activation by CKIP-1. Nat Cell Biol 10: 994–1002. 18641638 10.1038/ncb1760

[26] Ling S, Sun Q, Li Y, Zhang L, Zhang P, et al.. (2012) CKIP-1 Inhibits Cardiac Hypertrophy by Regulating Class II Histone Deacetylase Phosphorylation through Recruiting PP2A. Circulation. 10.1161/CIRCULATIONAHA.112.102780 23151343

[27] Wang Y, Nie J, Zhang L, Lu K, Xing G, et al (2012) CKIP-1 couples Smurf1 ubiquitin ligase with Rpt6 subunit of proteasome to promote substrate degradation. EMBO Rep 13: 1004–1011. 23032291 10.1038/embor.2012.144 PMC3492711

[28] Wang D, Matsumoto R, You Y, Che T, Lin XY, et al (2004) CD3/CD28 costimulation-induced NF-kappaB activation is mediated by recruitment of protein kinase C-theta, Bcl10, and IkappaB kinase beta to the immunological synapse through CARMA1. Mol Cell Biol 24: 164–171. 14673152 10.1128/MCB.24.1.164-171.2003 PMC303359

[29] Cvijic ME, Xiao G, Sun SC (2003) Study of T-cell signaling by somatic cell mutagenesis and complementation cloning. Journal of Immunological Methods 278: 293–304. 12957416 10.1016/s0022-1759(03)00191-1

[30] Kawano Y, Yoshida T, Hieda K, Aoki J, Miyoshi H, et al (2004) A lentiviral cDNA library employing lambda recombination used to clone an inhibitor of human immunodeficiency virus type 1-induced cell death. J Virol 78: 11352–11359. 15452256 10.1128/JVI.78.20.11352-11359.2004 PMC521860

[31] Yoshida T, Kawano Y, Sato K, Ando Y, Aoki J, et al (2008) A CD63 Mutant Inhibits T-cell Tropic Human Immunodeficiency Virus Type 1 Entry by Disrupting CXCR4 Trafficking to the Plasma Membrane. Traffic 9: 540–558. 18182005 10.1111/j.1600-0854.2007.00700.x

[32] Baier-Bitterlich G, Uberall F, Bauer B, Fresser F, Wachter H, et al (1996) Protein kinase C-theta isoenzyme selective stimulation of the transcription factor complex AP-1 in T lymphocytes. Mol Cell Biol 16: 1842–1850. 8657160 10.1128/mcb.16.4.1842 PMC231171

[33] Wertz IE, O'Rourke KM, Zhou H, Eby M, Aravind L, et al (2004) De-ubiquitination and ubiquitin ligase domains of A20 downregulate NF-kappaB signalling. Nature 430: 694–699. 15258597 10.1038/nature02794

[34] Gaide O, Favier B, Legler DF, Bonnet D, Brissoni B, et al (2002) CARMA1 is a critical lipid raft-associated regulator of TCR-induced NF-kappa B activation. Nat Immunol 3: 836–843. 12154360 10.1038/ni830

[35] Bromley SK, Burack WR, Johnson KG, Somersalo K, Sims TN, et al (2001) The immunological synapse. Annu Rev Immunol 19: 375–396. 11244041 10.1146/annurev.immunol.19.1.375

[36] Blonska M, Lin X (2009) CARMA1-mediated NF-kappaB and JNK activation in lymphocytes. Immunol Rev 228: 199–211. 19290929 10.1111/j.1600-065X.2008.00749.x PMC2878635

[37] Lenz G, Davis RE, Ngo VN, Lam L, George TC, et al (2008) Oncogenic CARD11 mutations in human diffuse large B cell lymphoma. Science 319: 1676–1679. 18323416 10.1126/science.1153629

[38] Ngo VN, Davis RE, Lamy L, Yu X, Zhao H, et al (2006) A loss-of-function RNA interference screen for molecular targets in cancer. Nature 441: 106–110. 16572121 10.1038/nature04687

[39] Lim KH, Yang Y, Staudt LM (2012) Pathogenetic importance and therapeutic implications of NF-kappaB in lymphoid malignancies. Immunol Rev 246: 359–378. 22435566 10.1111/j.1600-065X.2012.01105.x PMC4094296

[40] Brdicka T, Pavlistova D, Leo A, Bruyns E, Korinek V, et al (2000) Phosphoprotein associated with glycosphingolipid-enriched microdomains (PAG), a novel ubiquitously expressed transmembrane adaptor protein, binds the protein tyrosine kinase csk and is involved in regulation of T cell activation. J Exp Med 191: 1591–1604. 10790433 10.1084/jem.191.9.1591 PMC2213442

[41] Davidson D, Bakinowski M, Thomas ML, Horejsi V, Veillette A (2003) Phosphorylation-dependent regulation of T-cell activation by PAG/Cbp, a lipid raft-associated transmembrane adaptor. Mol Cell Biol 23: 2017–2028. 12612075 10.1128/MCB.23.6.2017-2028.2003 PMC149484

[42] Hayden MS, Ghosh S (2008) Shared principles in NF-kappaB signaling. Cell 132: 344–362. 18267068 10.1016/j.cell.2008.01.020

[43] Dobenecker MW, Schmedt C, Okada M, Tarakhovsky A (2005) The ubiquitously expressed Csk adaptor protein Cbp is dispensable for embryogenesis and T-cell development and function. Mol Cell Biol 25: 10533–10542. 16287865 10.1128/MCB.25.23.10533-10542.2005 PMC1291250

[44] Xu S, Huo J, Tan JE, Lam KP (2005) Cbp deficiency alters Csk localization in lipid rafts but does not affect T-cell development. Mol Cell Biol 25: 8486–8495. 16166631 10.1128/MCB.25.19.8486-8495.2005 PMC1265734

[45] Oneyama C, Hikita T, Enya K, Dobenecker MW, Saito K, et al (2008) The lipid raft-anchored adaptor protein Cbp controls the oncogenic potential of c-Src. Mol Cell 30: 426–436. 18498747 10.1016/j.molcel.2008.03.026

[46] Krishna S, Xie D, Gorentla B, Shin J, Gao J, et al (2012) Chronic activation of the kinase IKKbeta impairs T cell function and survival. J Immunol 189: 1209–1219. 22753932 10.4049/jimmunol.1102429 PMC3401317

[47] Lamason RL, Kupfer A, Pomerantz JL (2010) The dynamic distribution of CARD11 at the immunological synapse is regulated by the inhibitory kinesin GAKIN. Mol Cell 40: 798–809. 21145487 10.1016/j.molcel.2010.11.007 PMC3032410

[48] Bidere N, Ngo VN, Lee J, Collins C, Zheng L, et al (2009) Casein kinase 1alpha governs antigen-receptor-induced NF-kappaB activation and human lymphoma cell survival. Nature 458: 92–96. 19118383 10.1038/nature07613 PMC2688735

