# Supplementary figures and images for: CKIP-1 Is an Intrinsic Negative Regulator of T-Cell Activation through an Interaction with CARMA1
Source: PLoS One. 2014 Jan 17;9(1):e85762. doi: 10.1371/journal.pone.0085762 (PMC3894987; doi:10.1371/journal.pone.0085762)

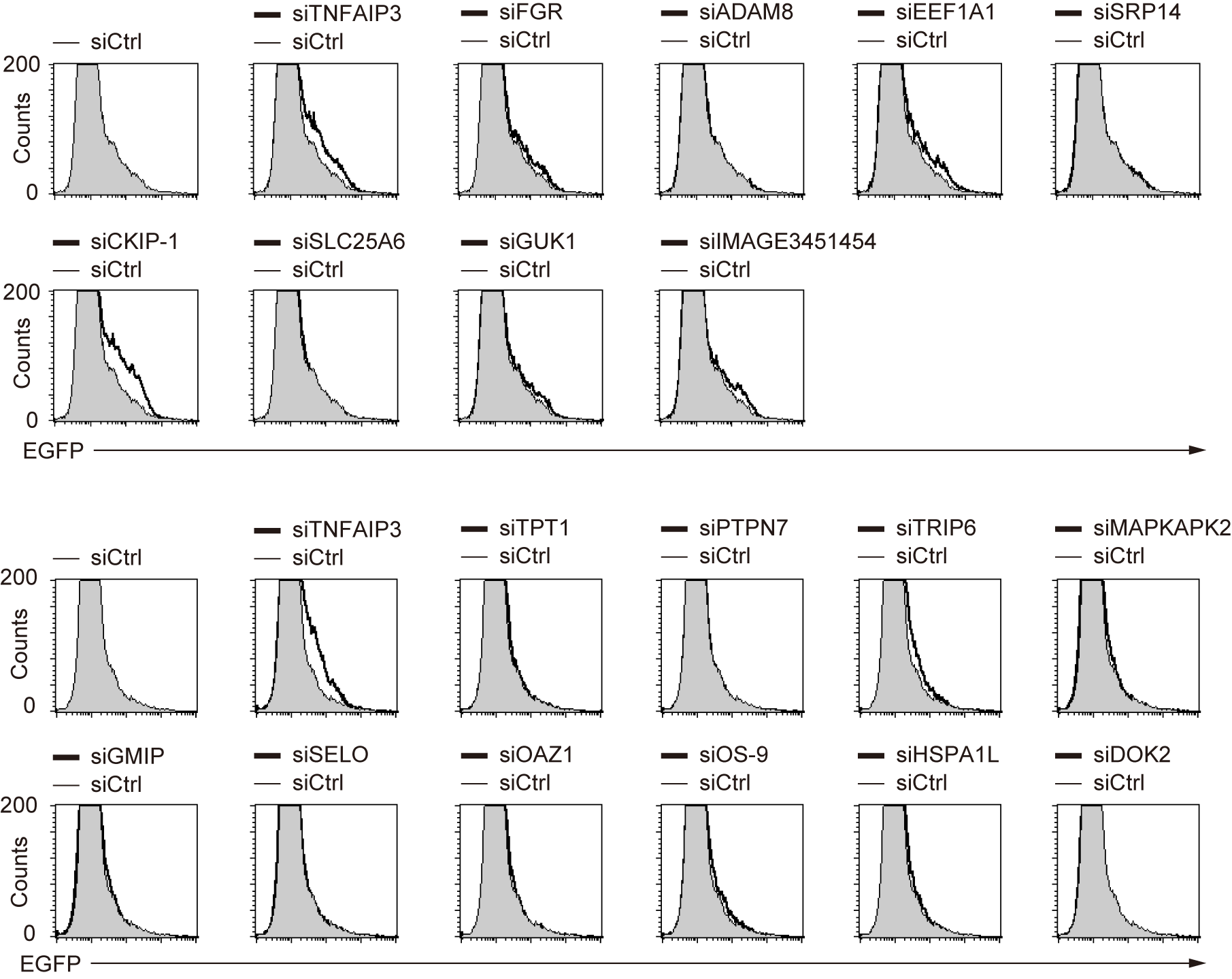

Supplement: Figure S1 — Knockdown of CKIP-1 induces NF-κB activation. The JR-GFP cells were electroporated with 400 pmol of non-targeting siRNA, or specific siRNA against each gene by AMAXA Nucleofector System. Five days later, the expression of EGFP was assessed by FACS. (TIF) [file pone.0085762.s001.tif]
